# Exposure-response modeling improves selection of radiation and radiosensitizer combinations

**DOI:** 10.1007/s10928-021-09784-7

**Published:** 2021-10-08

**Authors:** Tim Cardilin, Joachim Almquist, Mats Jirstrand, Astrid Zimmermann, Floriane Lignet, Samer El Bawab, Johan Gabrielsson

**Affiliations:** 1 Fraunhofer-Chalmers Centre, Chalmers Science Park, Gothenburg, Sweden; 2 Department of Mathematical Sciences, Chalmers University of Technology and University of Gothenburg, Gothenburg, Sweden; 3 Translation Innovation Platform Oncology, Merck KGaA, Darmstadt, Germany; 4 Translational Medicine, Merck KGaA, Darmstadt, Germany; 5 Meddoor AB, Gothenburg, Sweden; 6 Present Address: Clinical Pharmacology and Quantitative Pharmacology, Clinical Pharmacology & Safety Sciences, BioPharmaceuticals R&D, AstraZeneca, Gothenburg, Sweden

**Keywords:** Radiosensitizer, Tumor Static Exposure, Treatment optimization, Tumor growth model, Drug selection

## Abstract

A central question in drug discovery is how to select drug candidates from a large number of available compounds. This analysis presents a model-based approach for comparing and ranking combinations of radiation and radiosensitizers. The approach is quantitative and based on the previously-derived Tumor Static Exposure (TSE) concept. Combinations of radiation and radiosensitizers are evaluated based on their ability to induce tumor regression relative to toxicity and other potential costs. The approach is presented in the form of a case study where the objective is to find the most promising candidate out of three radiosensitizing agents. Data from a xenograft study is described using a nonlinear mixed-effects modeling approach and a previously-published tumor model for radiation and radiosensitizing agents. First, the most promising candidate is chosen under the assumption that all compounds are equally toxic. The impact of toxicity in compound selection is then illustrated by assuming that one compound is more toxic than the others, leading to a different choice of candidate.

**Supplementary Information:**

The online version contains supplementary material available at 10.1007/s10928-021-09784-7.

## Introduction

Radiotherapy is a cornerstone of modern oncology, and is frequently given in conjunction with chemical treatments to improve efficacy [1, 2]. Radiosensitizers are a class of chemical agents designed to enhance the radiation effect, e.g. by interfering with the cell’s repair of radiation-induced DNA damage [3]. During preclinical development of novel compounds, including radiosensitizers, a central question is how to select the most promising compounds from a large number of candidates [4–6]. Proper assessment of radiation and radiosensitizer combinations requires studies of efficacy as well as toxicology and adverse effects [7]. All compounds and doses cannot be tested in vivo—for reasons of time, resources, and ethics [8]. Experimental studies must therefore be supported by cheaper alternatives such as computer modeling and simulations [9, 10].

Numerous quantitative models have been developed to describe the effects of radiotherapy on tumors, with or without chemical intervention [11–14]. These models range from the simple, yet ubiquitous, linear-quadratic model of radiobiology [15], to complex systems pharmacology models that include particular pathways and processes (such as the cell cycle) that are relevant to the given treatment [16, 17]. In radiation oncology, models of Tumor Control Probability (TCP)—defined by whether a given radiation dose controls or eradicates an irradiated tumor—are commonly employed alongside Normal Tissue Complication Probability (NTCP) models that quantify toxicology and adverse risks [18–20].

Models have also been developed to describe the effects of radiotherapy on tumor volume over time. Watanabe et al*.* [21] proposed a radiation model with gradual cell death in response to single-dose treatment, and used it to describe tumor growth over time in rat rhabdomyosarcoma and in patients with metastatic brain tumors. More recently, Husband et al*.* developed and evaluated radiation models that describe tumor growth and survival in patient-derived xenograft mice for diffuse intrinsic pontine glioma [22].

In two earlier papers, we developed models that describe tumor growth in xenograft mice receiving radiotherapy and neoadjuvant radiosensitizing treatment [23, 24]. We also introduced the Tumor Static Exposure (TSE) concept—a model-based prediction of all combinations of radiation doses and radiosensitizer concentrations that result in tumor regression. However, these models only consider a single radiosensitzing compound and can therefore not fully illustrate the utility of the TSE concept in aiding the drug selection process.

In this paper, we use TSE to compare and rank three different combinations of radiation and radiosensitizing agents. One of our earlier models is used with data from a xenograft study involving radiotherapy administered alone or together with either of the radiosensitizers. The compounds are ranked by weighing efficacy (measured using TSE) against toxicity. Two different toxicological models are considered: a simple, linear model; and a more complex NTCP model adjusted to account for radiosensitizing treatment [25]. We also introduce the concept of Tumor Shrinkage Exposures, which can be used if tumor stasis is insufficient and tumor shrinkage with a particular rate is desired.

## Methods

Experimental data are first described. Then, a previously-developed tumor model used to describe radiation and radiosensitizer combination therapies is summarized. Thereafter, a method for comparing and ranking radiation and radiosensitizer combinations, based on TSE, is presented. The method optimizes a given cost function, used to describe, *e.g.,* toxicity and other adverse effects, along the TSE curve. Finally, computational aspects of the nonlinear mixed-effects modeling approach are provided.

### Experimental data

Pharmacodynamic data were generated in FaDu xenograft mouse models treated with radiation either alone or together with one of three early-discovery radiosensitizing compounds, henceforth referred to as compounds A_1_, A_2_, and A_3_. A total of 54 female mice were divided into six treatment groups with nine mice in each group: (A) vehicle control, (B) monotherapy with radiation (2 Gy per dose), (C) combination therapy with radiation (2 Gy per dose) and compound A_1_ (100 mg/kg per dose), (D) combination therapy with radiation (2 Gy per dose) and compound A_2_ (25 mg/kg per dose), (E) combination therapy with radiation (2 Gy per dose) and compound A_2_ (100 mg/kg per dose), and (F) combination therapy with radiation (2 Gy per dose) and compound A_3_ (20 mg/kg per dose). Doses were given once per day Mon–Fri for 6 weeks.

Exposure data were generated in FaDu xenograft models for the compounds A_1_, A_2_, and A_3_. Single doses of the compounds A_1_, A_2_, and A_3_ were given orally to 16 animals divided into four treatment groups with four mice in each group: compound A_1_ (100 mg/kg), compound A_2_ (25 mg/kg), compound A_2_ (100 mg/kg) and compound A_3_ (20 mg/kg) Drug concentration in plasma was measured after 1, 2, and 6 h.

Experiments were approved in accordance with German animal welfare regulations by the Regierungspräsidium Darmstadt, Hessen, Germany (protocol registration numbers DA 4/Anz. 397 and DA 4/Anz. 398).

### Tumor model for radiation and radiosensitizer combination treatment

**Fig. 1:**
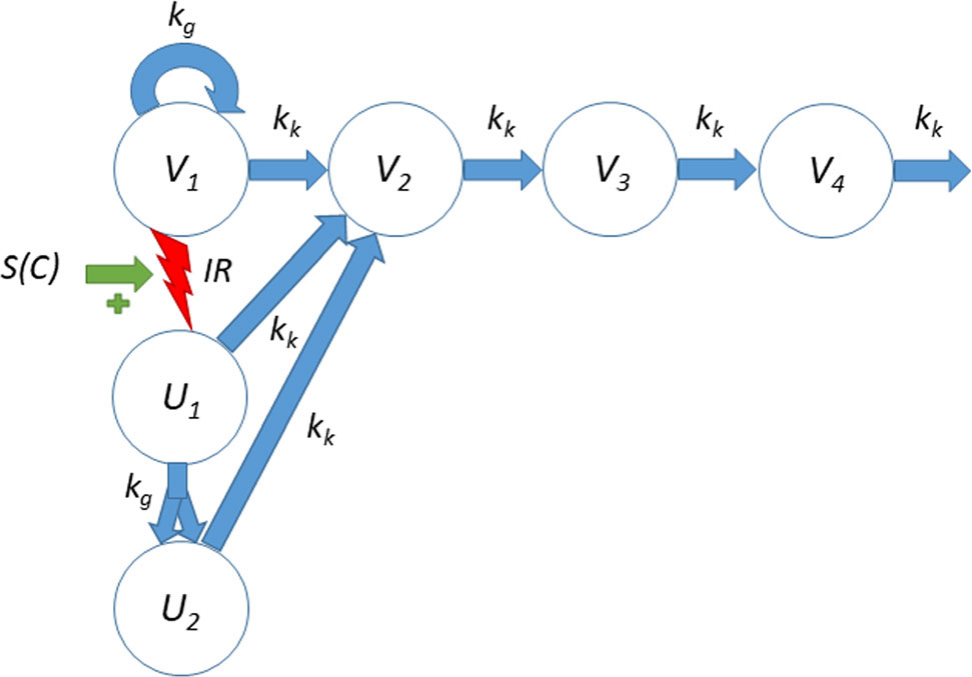
Tumor model used to describe combination therapy with ionizing radiation (IR) and radiosensitizer compounds. Cancer cells in compartment *V*_1_ proliferate with rate *k*_*g*_ and are eliminated with rate *k*_*k*_. Dying cells are transferred through three damage compartments *V*_2_, *V*_3_ and *V*_4_. Lethally irradiated cells are moved to a radiation-damage compartment *U*_1_ where they are allowed up to one more cell division, before dying. The compartment *U*_2_ represents irradiated cells after one cell division that can no longer divide

We use a previously-developed radiation model (Fig. 1) to describe tumor growth following treatment with radiation and radiosensitizing agents [23]. The model consists of a main compartment *V*_1_ representing proliferating cancer cells, three damage compartments *V*_2_, *V*_3_, and *V*_4_, that all dying cells traverse, and two radiation compartments *U*_1_, and *U*_2_, that allow irradiated cells up to one more cell division before dying. Irradiated cells are instantaneously transferred from *V*_1_ to *U*_1_. The fraction of proliferating cells that is transferred is based on the well-established linear-quadratic formula from radiobiology [14, 15]. Moreover, the presence of a radiosensitizing agent is accounted for via an increase in the number of lethally irradiated cells depending on the plasma concentration of the radiosensitizer at the time of irradiation. A high plasma concentration leads to a greater transfer of cells from *V*_1_ to *U*_1_. The model also incorporates natural cell death, meaning that some cells traverse the damage compartments even for untreated tumors.

The tumor model is described by the following differential equations

1 $$\begin{array}{*{20}l} {\frac{{dV_{1} }}{dt}} \hfill & { = k_{g} V_{1} - k_{k} V_{1} } \hfill \\ {\frac{{dV_{2} }}{dt}} \hfill & { = k_{k} V_{1} + k_{k} U_{1} + k_{k} U_{2} - k_{k} V_{2} } \hfill \\ {\frac{{dV_{3} }}{dt}} \hfill & { = k_{k} V_{2} - k_{k} V_{3} } \hfill \\ {\frac{{dV_{4} }}{dt}} \hfill & { = k_{k} V_{3} - k_{k} V_{4} } \hfill \\ {\frac{{dU_{1} }}{dt}} \hfill & { = - k_{g} U_{1} - k_{k} U_{1} } \hfill \\ {\frac{{dU_{2} }}{dt}} \hfill & { = 2k_{g} U_{1} - k_{k} U_{2} } \hfill \\ \end{array}$$

where *k*_*g*_ is the growth rate of proliferating cancer cells, and *k*_*k*_ the kill rate of cancer cells which is assumed to be the same for all compartments. The use of growth rate *k*_*g*_ and the presence of the factor two in the transfer from *U*_1_ to *U*_2_ describes that cell division occurs between these states and therefore twice as many cells enter *U*_2_ than leave *U*_1_.

Radiation treatment is implemented as sudden transfer between compartments *V*_1_ and *U*_1_, corresponding to an instantaneous transfer of cells with the fraction given by (1-*SF* (*D*_*IR*_,*C*_*i*_)). Here, *SF* (*D*_*IR*_,*C*_*j*_) is the surviving fraction of proliferating cancer cells given a radiation dose *D*_*IR*_ and concurrent drug plasma concentration *C*_*j*_ of compound A_j_. Mathematically this can be described by the two equations

2 $$\begin{array}{*{20}l} {V_{1} \left( {t_{i}^{ + } } \right)} \hfill & { = V_{1} \left( {t_{i}^{ - } } \right) - \left( {1 - SF\left( {D_{IR} \left( {t_{i} } \right),C_{j} \left( {t_{i} } \right)} \right)} \right)V_{1} \left( {t_{i}^{ - } } \right),} \hfill \\ {U_{1} \left( {t_{i}^{ + } } \right)} \hfill & { = U_{1} \left( {t_{i}^{ - } } \right) + \left( {1 - SF\left( {D_{IR} \left( {t_{i} } \right),C_{j} \left( {t_{i} } \right)} \right)} \right)V_{1} \left( {t_{i}^{ - } } \right),} \hfill \\ \end{array}$$

where *t*_*i*_ denotes the times of irradiation, and ${t}_{i}^{-}$ and ${t}_{i}^{+}$ can be interpreted as times just before and after irradiation. Note that radiation dose is given in terms of Gray (Gy), which is absorbed dose measured in joules per kilogram, *i.e.,* the radiation dose is normalized with respect to animal weight and hence plays the role of exposure to radiation. The surviving fraction is given by

3 $$SF\left( {D_{IR} ,C_{j} } \right) = \exp \left[ { - \left( {1 + a_{j} C_{j} } \right)\left( {\alpha D_{IR} + \beta D_{IR}^{2} } \right)} \right]$$

where *α* and *β* are the linear and quadratic coefficients associated with radiation DNA damage, and *a*_*j*_ is the pharmacodynamic parameter associated with the radiosensitizing capabilities of compound A_j_. The initial conditions for the system are given by

4 $$V_{i} \left( 0 \right) = V^{0} \left( {\frac{{k_{k} }}{{k_{g} }}} \right)^{i - 1} ,\,U_{i} \left( 0 \right) = 0,$$

where *V*^0^ is the initial volume of the main compartment. With these initial conditions, untreated tumors grow exponentially with net growth rate *k*_*g*_ − *k*_*k*_ [26]. The total tumor volume, *V*_total_, is obtained as the sum of all compartments

5 $$V_{{{\text{total}}}} = V_{1} + V_{2} + V_{3} + V_{4} + U_{1} + U_{2}$$

### Comparing combinations of radiation and radiosensitizers

In the case study, the goal is to select one of three radiosensitizing agents for further experimental study. We propose a model-based approach that evaluates combinations based on how easily tumor regression is achieved, relative to toxicological or other adverse effects. The model described in the previous section is calibrated to data and then used to derive TSE curves for each radiosensitizing agent. Cost functions are introduced to describe toxicology and other potential costs associated with treatment, and an optimization problem is formulated to minimize the cost subject to the constraint that the tumor does not grow, *i.e*., that the exposure is on or above the TSE curve.

The Tumor Static Concentration (TSC) and TSE concepts have been introduced and used in several earlier papers [26–29]. The TSC curve corresponding to a particular combination therapy consists of all pairs (C_1_, *C*_2_) of plasma concentrations for which a maintained exposure leads to tumor stasis. In particular, maintaining exposure levels above the TSC curve leads to tumor regression. The TSE concept is a generalization of TSC that allows for treatments for which concentrations are unknown or not applicable, such as radiotherapy. The TSE curve for the model given in Eqs. 1 and 2 has previously been derived (see [23]). The curve consists of combinations of daily radiation doses and average radiosensitizer concentrations such that the tumor is kept in approximate stasis.

In the Results section, the calibrated tumor model is used to generate TSE curves for combination therapy with radiation and each of three radiosensitizers, which we denote A_1_, A_2_, and A_3_. We propose the following procedure for comparing and ranking combinations, while accounting for toxicity and other adverse effects.

For each treatment combination, introduce an associated cost function Ψ (*E*_1_, *E*_2_), where *E*_1_ and *E*_2_ refer to general exposure metrics. In our case study *E*_1_ = *D*_*IR*_ is radiation dose, and *E*_2_ = *C*_*j*_ is the concurrent plasma concentration of radiosensitizer A_i_. Alternatively, nondimensional exposures could be defined by *E*_1_ = *D*_*IR*_/*D*_*ref*_ and *E*_2_ = *C*_*j*_/*C*_*ref,j*_, where *D*_*ref*_ and *C*_*ref,j*_ are reference exposures. The cost function is a way to measure the toxicity of the combination, although other kinds of costs could also be included. Ψ is an increasing function, reflecting that a larger value corresponds to higher cost/toxicity.

**Fig. 2:**
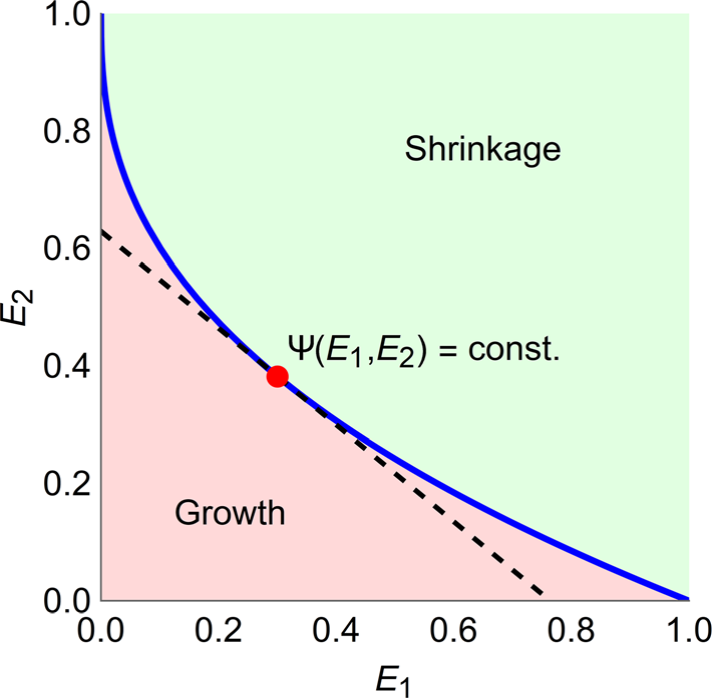
TSE curve for two compounds with exposures *E*_1_ and *E*_2_ (blue). Exposure pairs above the curve give rise to tumor shrinkage, whereas exposure pairs below the curve result in tumor growth. A level set where the cost Ψ is constant is shown in black, dashed, with the corresponding Ψ^*^ (color figure online)

In the simplest case, Ψ is linear function and is given by

6 $$\Psi \left( {E_{1} ,E_{2} } \right) = pE_{1} + qE_{2} ,$$

where *p*/*q* is the relative toxicity of the two compounds, assumed to be constant. Equation 6 assumes that toxicity increases linearly with exposure and is additive. Exposure pairs of equal costs, *i.e*., the level curves Ψ (*E*_1_, *E*_2_) = constant, are in this case lines with slope − *p*/*q*, with *E*_1_ and *E*_2_ are on the horizontal and vertical axes, respectively. An example of a TSE curve and a level set of the cost function is shown in Fig. 2.

As an example of a more intricate cost function, we utilize the established framework around NTCP [18, 19]. Such models are commonly used to describe the probability of adverse events following radiation treatment [30–32]. A widely used model for NTCP is the Lyman–Kutcher–Burman (LKB) model which defines the NTCP as

7 $$NTCP\left( t \right) = \frac{1}{{\sqrt {2\pi } }}\int_{ - \infty }^{t} {e^{{{{ - x^{2} } \mathord{\left/ {\vphantom {{ - x^{2} } 2}} \right. \kern-\nulldelimiterspace} 2}}} dx,}$$

where the variable *t* is defined by

8 $$t = \frac{{D_{eff} - TD_{50} }}{{mTD_{50} }},$$

and where *D*_*eff*_ is the effective dose, which accounts for non-uniform dose distribution, *TD*_50_ is the dose associated with 50% complication risk, and *m* is a slope parameter for the sigmoidal curve [25, 33]. From these equations we can see that a larger value of *D*_*eff*_ corresponds to a larger value for *t*, which in turns means a greater risk of complications.

The key question when defining a cost function for radiation and radiosensitizer combinations is how to introduce radiosensitizing treatment into the NTCP model. Since *TD*_50_ is a typical measure of radiation sensitivity, we propose to let the radiosensitizer modulate this parameter and thereby increase the risk of complications. Assuming an exponential sigmoidal modulation function gives a new definition of *t*,

9 $$t = \frac{{D_{eff} - TD_{50} I\left( C \right)}}{{mTD_{50} I\left( C \right)}}$$

where *I*(*C*) is an exponential inhibitory function with parameter *k*_*s*_ [34]:

10 $$I\left( C \right) = \exp \left( { - k_{s} C} \right).$$

We can thus use the NTCP model as a cost function with exposures *E*_1_ = *D*_*eff*_ = *D* (total radiation dose) and *E*_2_ = *C*, where *C* is the radiosensitizer concentration at the time of irradiation. Note that NTCP depends on the exposures *E*_1_ and *E*_2_ only through the variable *t*. Therefore, exposure combinations with equal complication risk have the same value for *t*. Thus, solving for *D*_*eff*_ in Eq. 9 gives the expression for equal cost

11 $$D_{eff} = \left( {1 + mt} \right)TD_{50} \exp \left( { - k_{s} C} \right).$$

Equation 10 describes a sigmoidal relationship between exposure pairs (*D*_*eff*_, *C*) with equal cost.

Equipped with a cost function, we search along the TSE curve for the exposure pair with the lowest cost. Repeating this procedure for each combination gives a sequence of lowest costs, each corresponding to a different combination therapy. These values can then be used to compare and rank combination therapies.

The procedure for comparing and ranking combinations is summarized below:

1. Choose a suitable tumor model given available data and calibrate the model to obtain parameter estimates
2. Compute the TSE curves and insert the estimated parameter values
3. Choose an appropriate cost function Ψ for each combination and find the minimum cost Ψ^*^ along the TSE curve
4. Repeat Steps 1–3 for each drug combination
5. Compare Ψ^*^ across combination therapies and choose the combination with the lowest cost

### Tumor shrinkage exposures

TSE curves are based on the requirement of tumor stasis. This is valuable since the curve divides the exposure plane into regions of tumor growth and tumor shrinkage. However, in practice, tumor shrinkage may not be enough and one can therefore look at tumor shrinkage with different rates. This leads to a generalization of TSE called tumor *shrinkage* exposure (TSE_*ρ*_) where *ρ* is the relative change in volume for a given time unit, $\rho = \frac{{V\left( {t_{1} } \right) - V\left( {t_{2} } \right)}}{{V\left( {t_{1} } \right)}}$, where *t*_2_ > *t*_1_ are two time point, and *t*_2_ − *t*_1_ is the chosen time unit. In particular, TSE_0_ is the regular TSE curve, and TSE_0.5_ requires that the tumor shrinks by 50% of its size every for every unit of time that elapses. TSE_*ρ*_ is derived analogously to TSE, with the difference that the growth rate is set to a constant different from zero. The concept of TSE_*ρ*_ curves is illustrated for the case study in the Results section.

### Computational methods

The tumor model was calibrated to xenograft data using a nonlinear mixed-effects approach based on the first-order conditional estimation (FOCE) method in a computational framework developed at the Fraunhofer-Chalmers Research Centre for Industrial Mathematics (Gothenburg, Sweden) and implemented in Mathematica (Wolfram Research) [35]. Exposure data for compounds A_1_, A_2_, and A_3_ were described using one-compartment pharmacokinetic models. Model evaluation was based on goodness-of-fit, empirical Bayes estimates, and residual analysis.

Lognormal distributed between-subject variability was added to the initial volume of the main compartment *V*_0_ and the growth rate *k*_*g*_. Residual errors were assumed to be proportional to tumor volume with zero mean and variance ${\sigma }_{V}^{2}$. As done previously, the ratio between *α* and *β* was set to 10 [23, 36].

## Results

First, the results of fitting the tumor model to the experimental data are presented. Then, TSE curves corresponding to combination therapy with radiation and each of the three radiosensitizers A_1_, A_2_, and A_3_, are computed. Finally, the procedure for comparing and ranking combinations is illustrated for two toxicological settings.

### Tumor model for radiation and radiosensitizer combination treatment

**Fig. 3:**
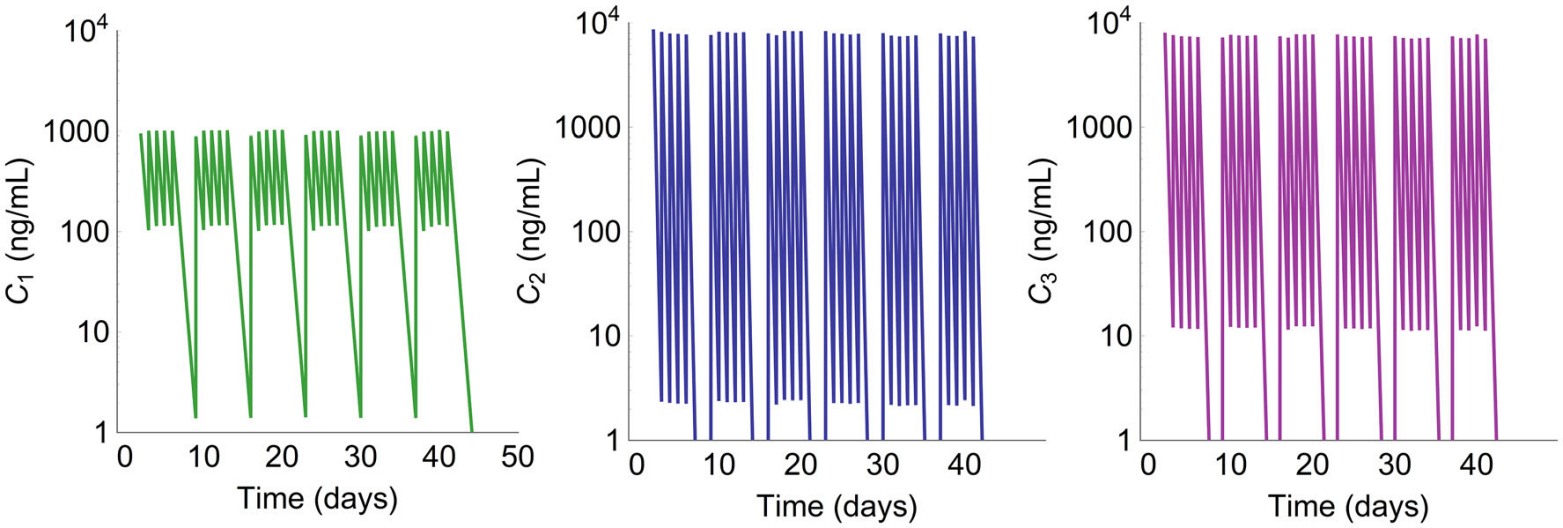
Simulated PK profiles for compounds A_1_, A_2_, and A_3_ with corresponding plasma concentrations *C*_1_, *C*_2_, and *C*_3_. Doses of 100 mg/kg (compounds A_1_ and A_2_) or 20 mg/kg (compound A_3_) were given 5 days a week for 6 weeks

Exposure profiles for each of the three radiosensitizers A_1_, A_2_, and A_3_ were described by standard one-compartment pharmacokinetic models, with parameter estimates given in Table 1. Simulated PK profiles used to drive the pharmacodynamic tumor model are shown in Fig. 3. The exposure of compound A_1_ (green) was approximately ten times lower than the exposures of compounds A_2_ (blue) and A_3_ (purple).

**Table 1:** Parameter estimates for the one-compartment pharmacokinetic models describing exposure to the compounds A_1_, A_2_, and A_3_ in terms of plasma concentration

Parameter	Compound	Population median (RSE%)	Between-subject variability^**a**^ (RSE%)
*k*_*e*_ (/h)	A_1_	0.092 (9)	63 (15)
	A_2_	0.35 (7)	18(13)
	A_3_	0.27 (9)	6 (13)
*V* (L/kg)	A_1_	110 (8)	26 (14)
	A_2_	12 (8)	14 (17)
	A_3_	2.6 (6)	2 (21)
$\sigma_{c}^{b}$(%)	A_1_	14 (25)	–
	A_2_	15 (22)	–
	A_3_	27 (20)	–

^a^*Calculated as *$$\sqrt {\omega_{ii}^{2} } \times 100$$

^b^*Intra-individual variability*

**Fig. 4:**
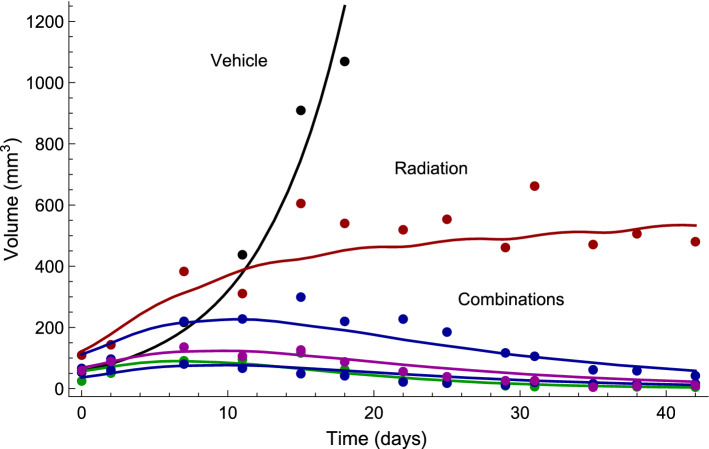
Examples of individual fits for each of the six treatment groups: vehicle (black), radiation monotherapy with 2 Gy per dose (red), combination therapy with radiation and A_1_ at 100 mg/kg per dose (green), combination therapy with radiation and A_2_ at 25 mg/kg or 100 mg/kg per dose (blue), and combination therapy with radiation and A_3_ at 20 mg/kg per dose (purple) (color figure online)

The tumor model adequately described observed data from each of the six treatment groups. Examples of individual fits for each treatment group are shown in Fig. 4. In the vehicle group, tumor growth was approximately exponential during the observed time period. Tumors treated with radiation monotherapy reached approximate stasis during treatment and in some cases showed signs of regression. Tumors treated with radiation and compound A_1_ combination therapy exhibited significant regression and in most cases the tumors were eradicated. Tumors treated with radiation and compound A_2_ showed significant regression with the lower dose (25 mg/kg) and in most cases tumor eradication with the higher dose (100 mg/kg). Lastly, tumors treated with radiation and compound A_3_ also exhibited tumor eradication in most instances. Visual predictive checks for the tumor model can be found in Supplemental Information S1.

The parameter estimates from fitting the tumor model simultaneously to all treatment groups are given in Table 2. The net growth rate $k_{g} - k_{k} = 0.16/{\text{day}}$ corresponds to an average doubling time of 4.3 days for the vehicle group. System and radiation parameters were estimated with good precision, whereas drug parameters were estimated with lower but still acceptable precision (RSE < 40%).

**Table 2:** Parameter estimates for the tumor model describing the effects of radiation and radiosensitizer combination therapy

Parameter	Population median (RSE%)	Between-subject variability^a^ (RSE%)	Description
*k*_*g*_ (/day)	0.50 (5)	53 (2)	Natural growth rate
*k*_*k*_ (/day)	0.34 (5)	–	Natural kill rate
V^0^ (mm^3^)	26.0 (8)	7 (11)	Initial volume of main compartment
*α* (/Gy)	0.11 (6)	–	Linear radiation parameter
β (/Gy^2^)	0.011 (6)	–	Quadratic radiation parameter
*a*_1_ (mL/μg)	0.27 (33)	–	Pharmacodynamic parameter of A_1_
*a*_2_ (mL/μg)	0.038 (36)	–	Pharmacodynamic parameter of A_2_
*a*_3_ (mL/μg)	0.028 (35)		Pharmacodynamic parameter of A_3_
$\sigma_{V}^{b}$(%)	28.0 (3)	–	Proportional standard error

^a^*Calculated as *$$\sqrt {\omega_{ii}^{2} } \times 100$$

^b^*Intra-individual variability*

### TSE curves for radiation and radiosensitizer combinations

Following the same principles as in [23] the following expression for the TSE_*ρ*_ curves was derived

12 $$D_{IR} = \frac{{ - \left( {\alpha + a_{j} \alpha C_{j} } \right) + \sqrt {\left( {\alpha + a_{j} \alpha C_{j} } \right)^{2} + 4\left( {\beta + a_{j} \beta C_{j} } \right)\left( {k_{g} T - k_{k} T + \log \left( {1 - \rho } \right)} \right)} }}{{2\left( {\beta + a_{j} \beta C_{j} } \right)}}$$

where *D*_*IR*_ is the radiation dose given every *T* days, and *C*_*j*_ is the plasma concentration of A_*j*_ at the instance of irradiation. The details can be found in Supplemental Information S2.

**Fig. 5:**
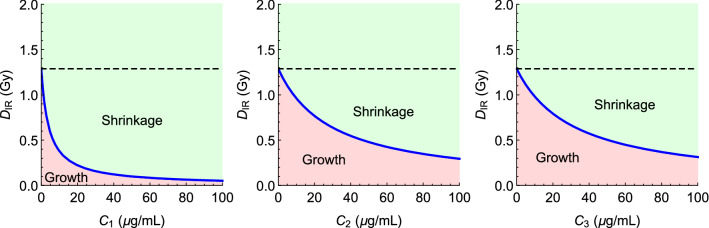
TSE curves for combinations of radiation and radiosensitizers A_1_ (left), A_2_ (middle) and A_3_ (right) obtained by inserting the parameter estimates from Table 2 into Eq. 12. TSE curves are shown in blue. Regions above and below the curves correspond to combination pairs that result in tumor shrinkage, or tumor growth, respectively. The dashed reference lines indicate the daily radiation dose required for tumor shrinkage during monotherapy

The TSE curves for the three combination therapies involving radiation and one of the radiosensitizing agents A_1_, A_2_, and A_3_, were computed by inserting the parameter estimates from Table 2 into Eq. 12, using *T* = 1 day to indicate daily dosing. The resulting TSE curves are shown in Fig. 5.

The TSE value for radiation monotherapy was estimated to 1.3 Gy, meaning that for the median individual a daily dose of 1.3 Gy would be sufficient for approximate tumor stasis. Since the compounds A_1_, A_2_, and A_3_, have no monotherapy effect, they have no TSE values. Instead, the TSE curves asymptotically approach the concentration axis as the plasma concentrations of the compounds approach infinity. The TSE curve for combinations of radiation and compound A_1_ (left) exhibits the largest curvature. Indeed, that TSE curve associated with A_1_ lies strictly below the TSE curve for the other two combination therapies.

### Comparing combinations of radiation and radiosensitizers

**Fig. 6:**
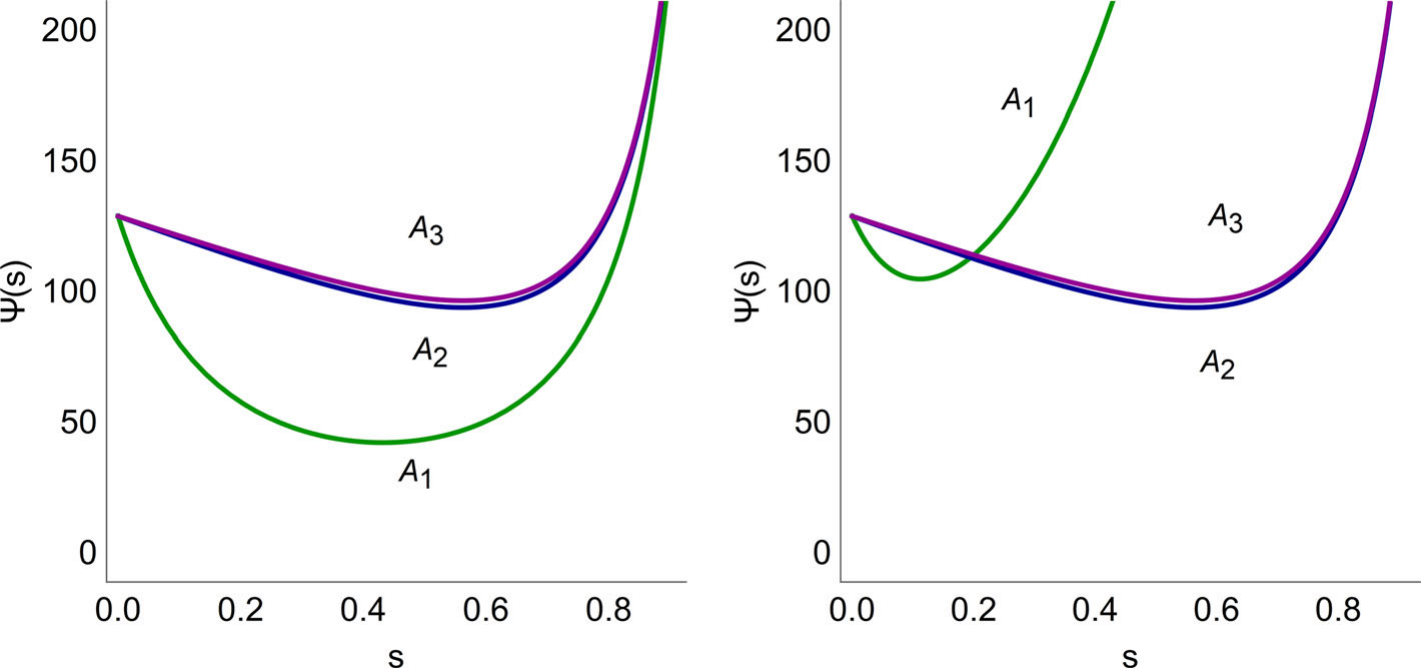
Hypothetical costs Ψ for different combinations along the TSE curves in Fig. 5 for combination therapy with radiation and radiosensitizers A_1_ (green), A_2_ (blue), and A_3_ (purple). The left plot assumes that all three compounds are equally toxic, whereas in the right plot the toxicity of A_1_ (green) has been increased by a factor ten. The parameter $\mathrm{s}$ represents the position on the TSE curve with *s* = 0 corresponding to radiation monotherapy and *s* = 1 monotherapy with compound A_*j*_ (color figure online)

The procedure outlined in the Methods section is applied to compare and rank the three combination therapies for two toxicological models. Using the first model, we consider two scenarios: one based on the assumption that all radiosensitizers are equally toxic, and one where the toxicity of compound A_1_ is increased tenfold. The cost functions are given by

13 $$\Psi \left( {D_{IR} ,C_{j} } \right) = pD_{IR} + q_{j} C_{j}$$

where *D*_*IR*_ is the radiation dose with toxicity coefficient *p*, and *C*_*j*_ is the plasma concentrations of compound *A*_*j*_ with toxicity coefficient *q*_*j*_. First, assuming that all test compounds are equally toxic means that *q*_1_ = *q*_2_ = *q*_3_. The costs associated with each combination pair (*D*_*IR*_, *C*_*j*_) on the corresponding TSE curve are illustrated in Fig. 6 (left). The parameter $s$ indicates the location along the TSE curve with *s* = 0 corresponding to radiation monotherapy, and *s* = 1 corresponding to monotherapy with the radiosensitizer. Note that, for the particular tumor model in this case study, the radiosensitizers have no monotherapy effect, which means that *s* = 1 corresponds to an infinitely large exposure of the radiosensitizer and therefore also an infinite cost/toxicity. The parametrization has been performed such that *s* = 0.5 corresponds to *C*_*j*_ = 25 μg/mL. This is an arbitrary scaling of the parametrization that does not affect the optimization problem and is performed only to make the figures easier to interpret. This amounts to the parametrization given in Eq. 14 below

14 $$\Psi \left( s \right) = pD_{IR} \left( {\frac{25s}{{1 - s}}} \right) + q_{j} \frac{25s}{{1 - s}}$$

with *D*_*IR*_ (*C*_*j*_) given as in Eq. 12.

The cost coefficients were set to *p* = 100/Gy and *q*_*j*_ = 1 mL/μg. Figure 6 (left) shows that combination therapy with A_1_ has the lowest cost (for *s* ≈ 0.5$$)$$. We then consider the second scenario, where the toxicity of A_1_ has been increased by a factor ten, *q*_1_ = 10 mL/μg, which is illustrated in Fig. 6 (right). A_1_ is no longer the best treatment option, since A_2_ and A_3_ both have lowers costs (occurring at *s* ≈ 0.6).

**Fig. 7:**
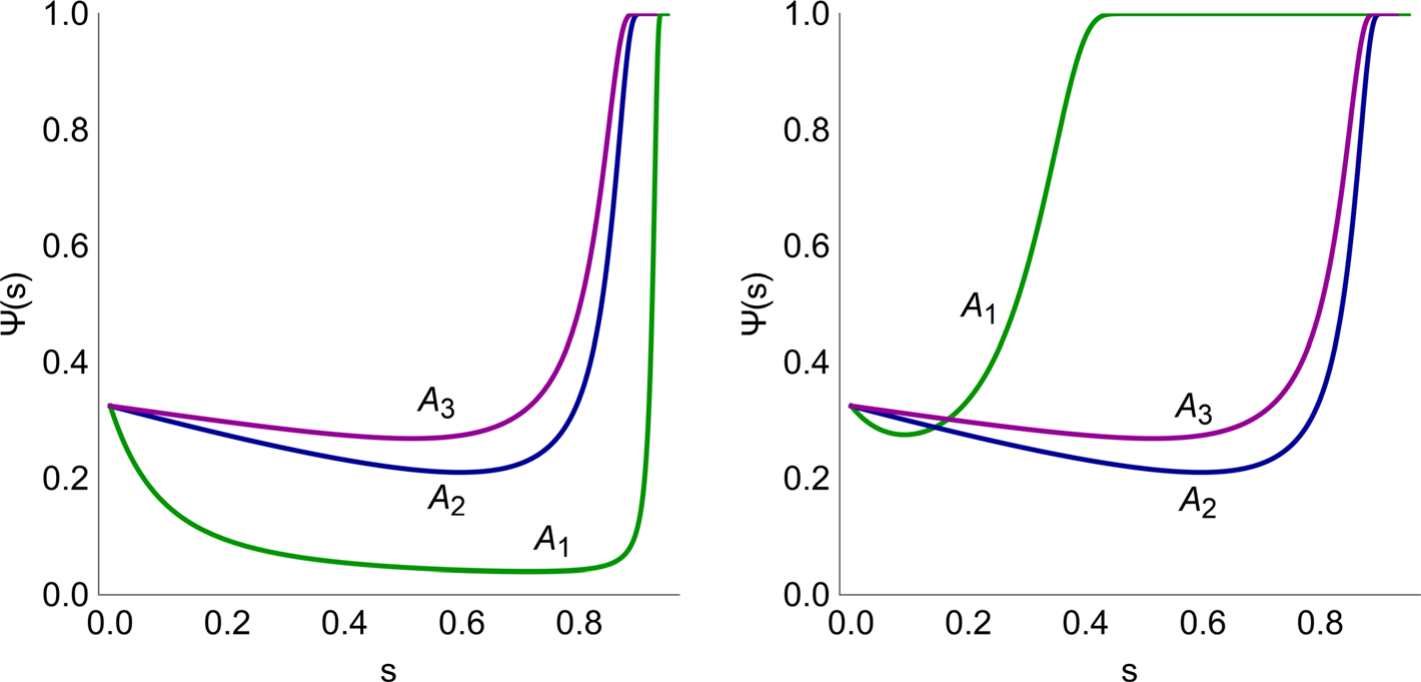
Hypothetical costs Ψ using the NTCP model (Eq. 15) for radiation and radiosensitizer combinations, A_1_ (green), A_2_ (blue), and A_3_ (purple), along the TSE curves in Fig. 5. The left plot assumes equal toxicity, whereas the right plot assumes a tenfold increase for radiosensitizer A_1_ (color figure online)

Figure 7 shows the results using the more complex NTCP model as cost function. For this model, we use values of *TD*_50_ = 50 Gy and *m* = 0.5 to describe radiation treatment and set the radiation parameter *k*_*s*_ to 0.02 mL/μg/day for all three radiosensitizers. Similar to the case with a linear cost function, A_1_, which is the most efficacious, has the lowest cost. Compared with the linear case, the value of the radiosensitizer parameter *k*_*s*_ for A_1_ would need to be decreased approximately tenfold for another radiosensitizer to become the most promising candidate. The parametrization of the cost function along the TSE curve, with parameter *s* going from *s* = 0 (radiotherapy) to *s* = 1 (radiosensitizer monotherapy) is given in Eq. 15.

15 $$\Psi \left( s \right) = NTCP\left( {\frac{{D_{IR} \left( {\frac{25s}{{1 - s}}} \right) - TD_{50} I\left( {\frac{25s}{{1 - s}}} \right)}}{{TD_{50} I\left( {\frac{25s}{{1 - s}}} \right)}}} \right)$$

where *D*_*IR*_ is given by Eq. 12, *I* is given by Eq. 10, and NTCP is given by Eq. 9.

### Tumor shrinkage exposures

**Fig. 8:**
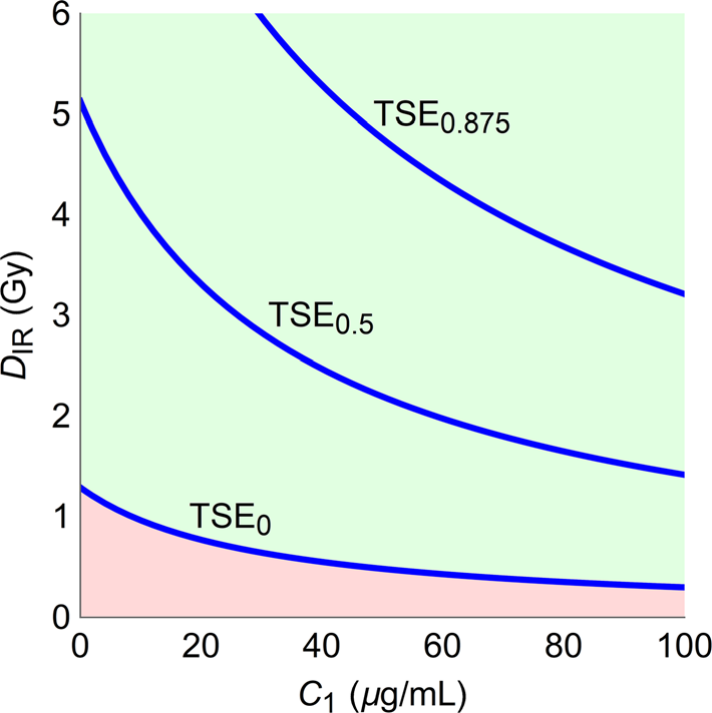
Examples of TSE curves for combinations of radiation and compound A_2_. TSE_1_, TSE_2_, and TSE_8_ corresponding to shrinkage rates that keep the tumor in stasis, reduce the tumor to half its size, and reduce the number of proliferating tumor cells to one eighth of its size with each daily dose, respectively

Figures 5 and 6 are used to minimize the cost of combination therapy with the respect to the TSE curve, *i.e.*, while making sure the tumor is not growing. As pointed out in the Methods section, it is also possible to require that the tumors shrink at a specified rate, by introducing the TSE_*ρ*_ curves. TSE_*ρ*_ curves are illustrated in Fig. 8 for combinations of radiation and compound A_2_, assuming a linear cost function as in Fig. 6. The three TSE_*ρ*_ curves (blue) consists of exposure pairs (*D*_*IR*_, *C*_*j*_) that keep the tumor in stasis, shrink the tumor to half its size, and shrink the tumor to one eighth of its size, respectively.

**Fig. 9:**
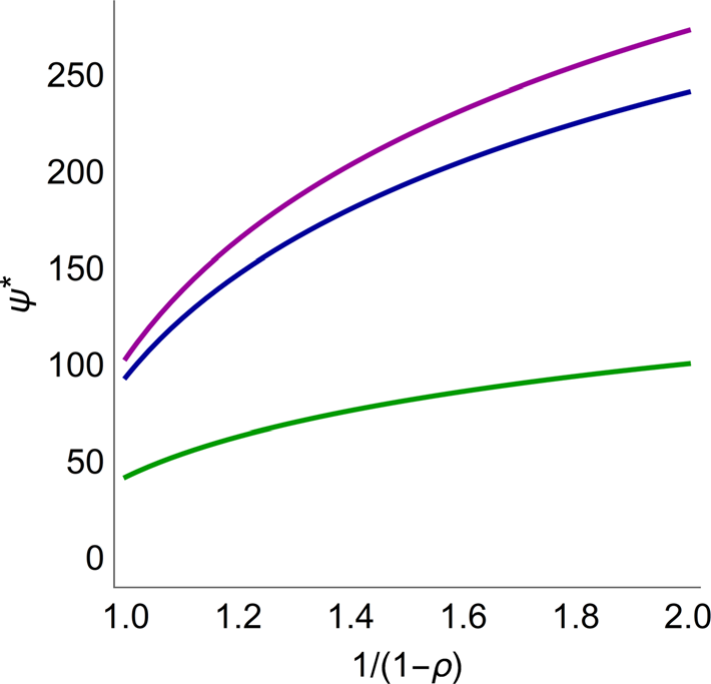
Minimal costs ψ^*^ as a function of relative shrinkage rate *ρ* for combinations of radiation and A_1_ (green), A_2_ (blue), and A_3_ (purple). Here $\frac{1}{1 - \rho } = {{V_{1} \left( {t_{1} } \right)} \mathord{\left/ {\vphantom {{V_{1} \left( {t_{1} } \right)} {V_{1} \left( {t_{2} } \right)}}} \right. \kern-\nulldelimiterspace} {V_{1} \left( {t_{2} } \right)}}$ is a more natural parameter such that the minimal cost is an increasing function of the parameter. The figure shows that, for any value of *ρ*, the compound A_1_ has the lowest cost, since the curves never intersect

For each TSE_*ρ*_ curve, it is possible to minimize the costs of combination therapy following the same procedure as described earlier. Thus, for a given combination, consider how the minimum cost, Ψ_min_, varies depending on how quickly the tumor is required to shrink, *i.e.*, *ρ*. Figure 9 depicts this scenario for combinations of radiation and the three radiosensitizers as a function of the parameter ${1 \mathord{\left/ {\vphantom {1 {1 - \rho }}} \right. \kern-\nulldelimiterspace} {1 - \rho }}$ under the assumption that all compounds are equally toxic. Note that combination therapy with A_1_ (green) always has the lowest cost.

## Discussion

Recent decades have seen a growing focus on combination therapies as a way to combat resistances and to obtain synergistic effects [37, 38]. Our analysis of radiotherapy and radiosensitizer combinations in this paper is focused on the latter, while also addressing toxicity and side-effects. As with any model-based analysis, good predictions and results are contingent not only on data [39], but also on sound modeling methodology [40, 41]. Details on this topic, particularly in the context of oncology, can be found *e.g.* in several papers by Mould et al*.* [42–44]. The remainder of this discussion considers, in order: the mathematical tumor model, the resulting TSE curves and concepts, and, finally, our proposed optimization and ranking procedure for radiation and radiosensitizer combinations.

### Tumor model for radiation and radiosensitizer combinations

The model used in this paper in based on an earlier model (see [23]), with two minor differences. As in [24] an exponential growth function was favored over logistic growth, since it proved sufficient to describe vehicle data and no plateaus in tumor volumes were observed. Secondly, we assumed no monotherapy effect for the radiosensitizers, which is expected to be negligible given that the compounds interact with the repair mechanisms of DNA damage induced by irradiation.

The growth and kill rates were estimated to similar values to those in [23, 24], and the net growth rate *k*_*g*_ − *k*_*k*_ of 0.15/day, corresponds to a doubling time of 4–5 days, which is similar to other models [21, 23, 26, 27, 45]. The estimated *α* and *β* values of 0.11/Gy and 0.0011/Gy^2^ are in line with reported ranges of 0.02 − 0.2 for *α* and 0.001 − 0.6 for *β* [46]. Model parameters were estimated with reasonable precision, although the radiosensitizer parameters *a*_*i*_ had somewhat lower precision (RSE% ≈ 35), which is partially explained by the fact that each *a*_*i*_ is only informed by one or two treatment groups, whereas other model parameters are informed by all data.

Like many tumor models used preclinically, our model contains a sequence of damage compartments [26, 27, 29, 45], and can be viewed as a combination of these models with the linear-quadratic model in radiobiology [15], or compartment radiation models that implement the linear-quadratic model with delay [21, 22]. Compared with systems pharmacology models for radiation and chemical combinations, *e.g.,* Checkley et al. [17], Kosinsky et al. [16], our model is simpler and, although less mechanistic, can be calibrated to standard xenograft data.

### TSE curves for radiation and radiosensitizer combinations

TSC and TSE have been developed and applied in a series of papers [23, 26, 27, 29]. They are tailored specifically to cancer treatments (single-agents or combinations), and are connected to qualitative behavior (tumor growth or shrinkage) of the disease as well as synergy, unlike general models such as the isobologram [47, 48] and the half-maximal effect curve [49] which focus only on synergy. In radiation oncology, TCP models are used to assess probabilities of tumor eradication, recurrence, or emergence of metastases [20, 50], which is similar to TSE in that it also aims to control or destroy cancer cells.

In our analysis, greatest synergy occurred with radiosensitizer A_1_, which can also be seen from the curvatures of the TSE curves (Fig. 5). This happens because although observed tumor growth was similar across combinations, exposure levels were approximately ten times lower for compounds A_1_ (see Fig. 3). However, proper assessment requires consideration not only of efficacy, but joint consideration of efficacy and toxicity.

### Comparing combinations of radiation and radiosensitizers

In our case study involving radiation and three radiosensitizing agents, Fig. 6 (left) shows that radiosensitizer A_1_ is the superior radiosensitizer given that all compounds are equally toxic, which holds for either cost function. Moreover, Fig. 6 (right) shows that the toxicity of A_1_ would have to be increased tenfold over A_2_ and A_3_ for another radiosensitizer to become preferable. This result held true for both cost functions. However, since the NTCP model is nonlinear and contains multiple sigmoidal functions, these results depend on the chosen parameter values.

Our proposed method evaluates combinations of radiation and radiosensitizers by the ability to induce tumor regression relative to toxicity. Two toxicity functions, or cost functions are considered: one linear, and one based on NTCP. The former approach was also considered in [29] to find an optimal combination for two anticancer compounds. A similar analysis can be found in [51] where phase one clinical data were used to construct a toxicity function with linear terms as well as a quadratic term to penalize combination treatment.

In radiotherapy, TCP and NTCP models are often combined to optimize treatment [52, 53]. In our analysis, the tumor model together with TSE appear instead of a TCP model, which we consider in conjunction with the commonly used Lyman NTCP model [25]. Here, we note similar results using a linear model and an NTCP model (see Figs. 6, 7) although the sigmoidal nature of the NTCP model produced somewhat flatter cost around the minima, which implies that good therapeutic response is less sensitive to perturbations and is therefore easier to achieve. Alternative NTCP models also exist (see e.g., [54–57]) although most tend to be static (as opposed to dynamic, or temporal) and empirically founded.

Dynamic models of toxicity have also been developed. In [58] Krzyzanski et al*.* proposed a model of thrombocytopenia following combined chemotherapy and radiation treatment. Scenarios when the tumor model as well as the toxicity model are both dynamic can be approached using optimal control theory [59, 60]. The approach to selecting and ranking combinations presented in this paper could also be used in combination with other optimization approaches such as those that design treatment protocols to yield the most amount of information about the compounds [61, 62].

### Conclusions and perspectives

We have demonstrated how a model-based approach, using TSE, can be used to compare and rank radiation and radiosensitizer combinations. The analysis weighs efficacy (tumor regression) against side-effects (toxicity) in order to provide a fair comparison and ranking of the different combinations.

While the chosen criteria for comparing combination therapies are natural, they are not the only reasonable choice. An alternative choice could be to compare the rate of tumor regression for each combination at a specified maximum tolerable exposure, *i.e.,* exchanging the roles that efficacy and toxicity play in the optimization problem.

Our analysis is focused on radiotherapy combined with radiosensitizing treatment. A similar approach using TSE and cost functions could also be considered for chemical combinations. However, the underlying pharmacokinetic, pharmacodynamic, and toxicity modeling would have to account for potential drug interactions.

## Supplementary Information

Below is the link to the electronic supplementary material.

Supplementary file1 (DOCX 136 KB)

Supplementary file2 (DOCX 15 KB)
