# Supplementary material for: Exposure-response modeling improves selection of radiation and radiosensitizer combinations
Source: J Pharmacokinet Pharmacodyn. 2021 Oct 8;49(2):167–78. doi: 10.1007/s10928-021-09784-7 (PMC8940791; doi:10.1007/s10928-021-09784-7)
Supplement: Supplementary file 1 — Supplementary file1 (DOCX 136 KB) [file 10928_2021_9784_MOESM1_ESM.docx]

**Supplemental Information S1**

1000 hypothetical individuals were simulated for each treatment group based on the tumor model (Eqs. 1 and 2) and the parameter estimates from Tables I and II. The resulting visual predictive checks comparing simulated 10^th^, 50^th^, and 90^th^ percentiles with observed tumor volumes are shown in Fig 10. Model predictions are in relatively good agreement with data, with the exception of two outlier individuals, shown in orange and purple, in the third and sixth VPCs, respectively.

**
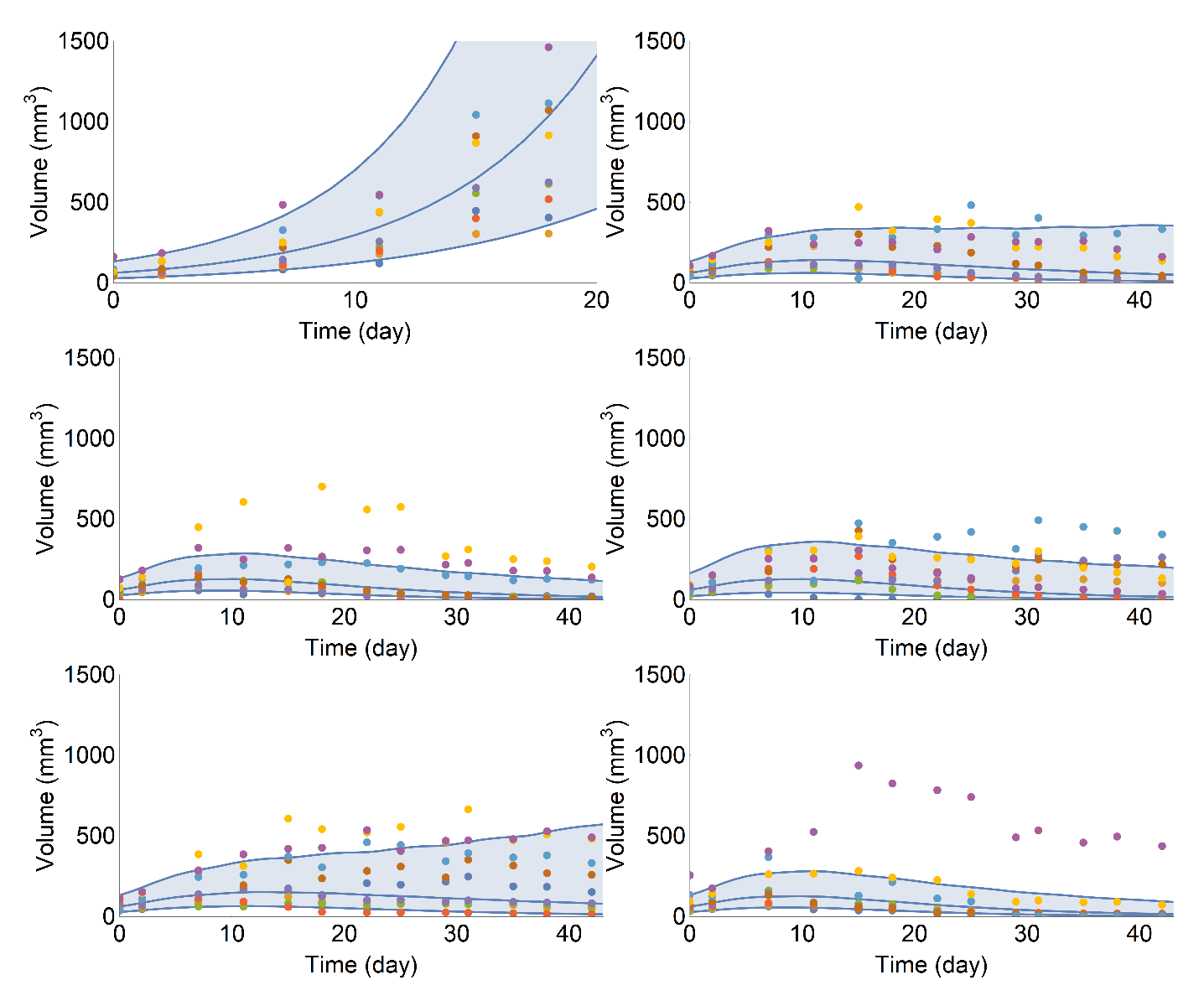
**

*Figure 10: Visual predictive check for the tumor model in Eqs. 1 and 2 using the parameter estimates from Tables I and II. Solid lines represent the simulated 10^th^, 50^th^ and 90^th^ percentiles. Colored dots represent observations. VPCs correspond to treatment groups in the following order from top left to bottom right: vehicle, radiation, radiation +* $A_{1}$ *(100 mg/kg), radiation +* $A_{2}$ *(25 mg/kg), radiation +* $A_{3}$ *(100 mg/kg), and radiation +* $A_{3}$ *(20 mg/kg).*
