# Supplementary material for: Exposure-response modeling improves selection of radiation and radiosensitizer combinations
Source: J Pharmacokinet Pharmacodyn. 2021 Oct 8;49(2):167–78. doi: 10.1007/s10928-021-09784-7 (PMC8940791; doi:10.1007/s10928-021-09784-7)
Supplement: Supplementary file 2 — Supplementary file2 (DOCX 15 KB) [file 10928_2021_9784_MOESM2_ESM.docx]

**Supplemental Information S2**

This document presents a derivation of the TSE expression in Eq. 12. The derivation closely resembles the one presented earlier in [23].

Assume that doses of radiation and radiosensitizer are given every $T$ days. Find a condition that ensures a given relative change of the main compartment, $V_{1}$, between times $t=0$ and $t=T$, where $T$ denotes the time between doses. The fraction of cells that survive irradiation is given by $SF\left( D,C_{i} \right)$, where $C_{i}$ denotes the plasma concentration of A_i_ at the time of irradiation. Until the time of the next dose of radiation, Eq. 1 implies that this fraction grows according to

|  | ${\frac{{dV}_{1}}{dt}=k}_{g}V_{1}-k_{k}V_{1}, V_{1}\left( 0 \right)=V_{0} SF(D,C)$ | $(16)$ |
| --- | --- | --- |

which has the solution

|  | $V_{1}\left( t \right)=V_{0} SF(D,C_{i})\exp\left( (k_{g}-k_{k})t \right)$ | $(17)$ |
| --- | --- | --- |

The relative change in proliferating cells $\rho=\frac{V_{0}-V_{1}(T)}{V_{0}}$ can then be computed using Eq. 3 and taking the logarithm, which gives the condition

|  | $\left( 1+a_{i}C_{i} \right)\left( \alpha D+\beta D^{2} \right)-{(k}_{g}-k_{k})T=-log(1-\rho)$ | $(18)$ |
| --- | --- | --- |

Eq. 18 can be viewed as a quadratic equation in $D$ that can be solved by standard formulas. The solution is given by

|  | $D=\frac{-\left( \alpha+a_{i}\alpha C_{i} \right)\pm\sqrt{{(\alpha+a_{i}\alpha C_{i})}^{2}+4\left( \beta+a_{i}\beta C_{i} \right)\left( k_{g}T-k_{k}T-\log(1-\rho) \right)}}{2\left( \beta+a_{i}\beta C_{i} \right)}$ | $(19)$ |
| --- | --- | --- |

where only the positive solution is biologically relevant. The individual TSE value corresponding to treatment only with radiation can be found by setting $C_{i}=0$ in Eq. 19.
